# Mode I Debonding Characterisation in Polymer-Based Sandwich Structures: A Review of Experimental Methods

**DOI:** 10.3390/polym18121512

**Published:** 2026-06-17

**Authors:** Amal Alliyankal Vijayakumar, Francesca Lionetto, Alfonso Maffezzoli

**Affiliations:** Department of Engineering for Innovation, University of Salento, Via per Monteroni, 73100 Lecce, Italy; francesca.lionetto@unisalento.it (F.L.); alfonso.maffezzoli@unisalento.it (A.M.)

**Keywords:** polymer sandwich structure, mode I characterisation, single cantilever beam, climbing drum peel, double cantilever beam, skin/core debonding, interfacial failure

## Abstract

Polymer-based sandwich structures are widely used for their lightweight and tailorable properties, but interfacial failure phenomena often govern their performance. Among these, Mode I skin/core debonding is a critical mechanism that limits structural reliability. This review provides a unified and critical assessment of experimental methodologies for Mode I fracture characterisation, focusing on the ASTM D8637/D8637M standard and alternative setups, including Double Cantilever Beam (DCB), Single Cantilever Beam (SCB), and Climbing Drum Peel (CDP) tests. Alongside the influence of geometrical factors, processing conditions and intrinsic polymer properties on Mode I characterisation are detailed. Conventional DCB setups are shown to introduce mixed-mode effects due to asymmetric loading. In contrast, the modified DCB-UBM setup achieves near-pure Mode I conditions at the expense of increased complexity. Comparative analysis indicates that the SCB configuration with a roller base outperforms the standardised flexible-rod setup, particularly for specimens with non-linear responses. The review also indicates that Mode I debonding behaviour is strongly influenced by several factors, including interfacial adhesion quality, constituent material properties, manufacturing-induced defects, specimen configurations, and environmental factors. Therefore, the interpretation of debonding performance requires a comprehensive structure–property–processing framework. Moreover, geometric constraints imposed by ASTM D8637/D8637M are also revisited, demonstrating that reduced-dimension specimens can yield comparable fracture toughness, thereby enabling greater design flexibility. Additionally, while the standard prescribes Modified Beam Theory (MBT) and Area Method (AM) for initiation and propagation, both methods provide comparable propagation toughness under linear conditions. For non-linear systems, alternative data reductions based on CDP concepts, with the SCB–roller base setup, are effective. Based on this assessment, key challenges and potential improvements are identified, guiding the development of more accurate and reliable testing methodologies for polymer sandwich structures.

## 1. Introduction

Advances in polymer processing technologies, encompassing both thermoplastic and thermoset systems, together with improvements in manufacturing efficiency, are driving the widespread adoption of polymer-based sandwich composites across diverse engineering applications [1]. In such systems, the intrinsic behaviour of the polymer matrix, including viscoelasticity, ductility, and temperature-dependent response, plays a critical role in governing structural performance and failure. Consequently, a thorough understanding of the failure mechanisms governing polymer-based sandwich structures is essential to ensure safe and reliable service.

Polymer-based sandwich structures, consisting of fibre-reinforced skins bonded or welded to lightweight cores, exhibit complex failure mechanisms [2,3,4,5]. While thermoset-based systems generally display relatively brittle behaviour, thermoplastic matrices can exhibit significant plastic deformation and rate-dependent response, leading to distinct fracture characteristics. The most significant failure modes, according to Zenkert et al. [6], are: skin failure, skin wrinkling, core shear failure, localised core crushing, global buckling and skin/core interface debonding. Among different damage modes, debonding at the skin/core interface is a crucial failure mechanism in polymer-based sandwich panels [3,7,8]. Experimental and numerical studies indicate that interfacial failure is the dominant failure mechanism as insufficient adhesion between the skin and core compromises the structural integrity and load-transfer capability of the entire panel [4,7,9,10,11,12]. Unlike skin or core failures, this mode is strongly influenced by the manufacturing process, leading to insufficient skin/core wetting, particularly at welded or co-consolidated interfaces, where incomplete fusion, voids, or thermal gradients may be present [7,13,14]. The sensitivity of polymers to temperature, strain rate, and time-dependent viscoelastic effects further contributes to interfacial degradation during service. Other factors that contribute to the failure mode include service temperature and in-service loading over a period of time due to overloading and impact-initiated debonds [15,16,17,18]. Debonding has been linked to various in-service failures, including structural issues in rudders and malfunctions of other aerospace control surfaces [19,20]. Its importance lies in the fact that it often remains barely visible while drastically reducing load transfer efficiency, promoting premature face wrinkling or core collapse. Hence, careful attention must be given to interface design, welding or bonding quality, and damage tolerance assessment with a high level of reliability to ensure safe and dependable performance of sandwich structures across their full operational load envelope.

Skin/core debonding occurs when the skin separates from the core in a sandwich structure, which can compromise the structural integrity of the component. Therefore, the strength of a skin/core interface can be quantified by measuring the energy required to separate the skin from the core, commonly referred to as the interfacial fracture toughness or critical strain energy release rate (SERR) [8,21,22,23]. Understanding damage tolerance is essential in the design and analysis of sandwich structures as it involves identifying and predicting crack initiation and propagation. Although standardised fracture toughness testing methods are well established for composite laminates, sandwich composites have historically received much less attention, with formal test protocols for failure modes emerging only in recent years. Skin/core interfacial debonding in sandwich structures can occur in various modes, including debonding under Mode I (opening), Mode II (shear) and mixed-mode (combination of Mode I and Mode II) conditions, each necessitating specific characterisation approaches.

For skin/core interfaces, fracture toughness must be characterised, in principle, over a range of mixed-mode conditions (Mode I + Mode II). Cracks in service are generally subjected to combined opening (Mode I) and shear (Mode II) loading rather than a single pure mode [24,25]. Nevertheless, most numerical and experimental studies focus on dominant Mode I rather than Mode II or mixed-mode loading conditions [8,21,22,23,26,27,28,29,30,31]. This is because Mode I is the most critical and governing fracture mode for skin/core debonding. Ratcliffe et al. [23] and many other researchers [11,32,33] also claimed that the most critical failure mode at the skin/core crack tip is Mode I. Moreover, in several material systems, the Mode I fracture toughness of skin/core interfaces is typically much lower than the corresponding Mode II, which further promotes Mode I-controlled crack initiation [26,28]. Accurate characterisation of Mode I interfacial fracture toughness is therefore essential for predicting failure, improving design, and ensuring structural safety. Several experimental methodologies have been developed [8], among which ASTM D8637/D8637M [34] has recently been used for evaluating Mode I skin/core debonding resistance. The standard provides guidelines for specimen geometry and data reduction based on beam-theory assumptions, aiming to ensure that dominant Mode I conditions are maintained during testing of sandwich systems under linear loading.

Alternative and modified testing approaches, including adaptations of the double cantilever beam (DCB) [35,36,37,38,39], single cantilever beam (SCB) [22,23,38,40,41,42,43] and peel-test (PT) methods [8,29], were widely used for measuring Mode I in polymer-based sandwich panels. These techniques aim to replicate better dominant Mode I conditions and improve the reliability of fracture toughness measurements. Wiedmann et al. [8] presented a comprehensive review of various Mode I characterisation techniques in thermoplastic sandwich panels, analysing methods such as DCB, SCB, and peel tests for evaluating dominant Mode I conditions at crack tips. However, a systematic and critical comparison of these approaches with the current standard, ASTM D8637/D8637M [34], is still lacking. In particular, recent advancements in test configurations, such as DCB-UBM and SCB adapted for non-linear loading, as well as emerging sizing methodologies in sandwich structures for Mode I testing, have not yet been collectively assessed within a unified framework. Moreover, the coupled influence of data reduction and geometrical-material parameters on fracture behaviour and measurement reliability remains insufficiently understood.

**Figure 1:**
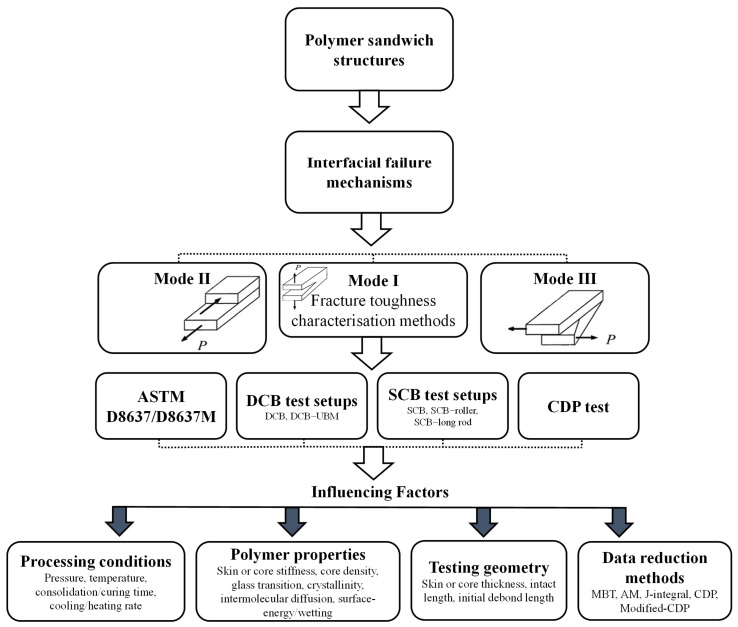
Schematic block diagram illustrating the organisation of the present review.

To address these gaps, this review provides, for the first time, a unified and critical assessment of experimental methods for Mode I characterisation in polymer-based sandwich structures, with a specific focus on the limitations of ASTM D8637/D8637M [34]. Unlike previous studies, this work systematically integrates and compares conventional and emerging test configurations while explicitly evaluating the influence of data reduction methods, key geometrical-material parameters and processing condition (such as curing or polymeric chain interdiffusion) relationships on fracture toughness measurements. Furthermore, it highlights the limitations of current standardised approaches under non-linear and realistic service conditions. The pictorial representation of the organisational framework of the review is shown in Figure 1. Based on this comprehensive analysis, the review identifies critical challenges and proposes targeted recommendations for improving testing methodologies, thereby contributing to the advancement of more accurate, reliable, and application-relevant standards for Mode I characterisation of sandwich structures.

## 2. Literature Search and Selection Strategy

A structured literature search was conducted to identify studies related to Mode I interfacial fracture characterisation of sandwich structures. The primary databases consulted included Scopus, Web of Science, Google Scholar and relevant ASTM standards. These sources were chosen for their broad coverage of polymer-based sandwich structures, fracture mechanics and standardised testing methodologies. The search employed combinations of keywords, including “Mode I fracture”, “interfacial fracture toughness”, “sandwich structures”, “skin/core debonding”, “double cantilever beam (DCB)”, “single cantilever beam (SCB)”, “climbing drum peel (CDP)”, and “ASTM D8637”.

The review focused on peer-reviewed journal articles, ASTM standards, and key technical publications relevant to the experimental characterisation of Mode I fracture behaviour in sandwich structures. Studies were included if they investigated Mode I interfacial fracture behaviour, fracture toughness evaluation, test method development, specimen design requirements, data reduction techniques, or the influence of material and geometrical parameters on fracture response. Studies primarily focused on Mode II or Mode III fracture behaviour or monolithic composite laminates without sandwich construction and publications lacking sufficient experimental details were excluded from the detailed analysis.

The literature search covered publications from 1990 to 2026, ensuring inclusion of both foundational works and recent advancements in sandwich composite fracture mechanics. Particular emphasis was placed on recent publications, with approximately 60–70% of the selected studies published within the last 10–15 years (2011–2026), reflecting the growing research focus on advanced thermoplastic sandwich structures, improved testing methodologies, and environmental durability effects. However, seminal studies and standards were retained due to their continued relevance in defining fracture mechanics frameworks and experimental procedures. Therefore, foundational studies and standards were included alongside recent publications to provide a comprehensive and balanced assessment of Mode I fracture behaviour in sandwich structures. The selected literature was subsequently categorised according to test configuration, loading conditions, fracture behaviour, specimen sizing requirements, and data reduction methods to facilitate a critical comparison of the available approaches.

## 3. Mode I Skin/Core Interfacial Debonding Methods

**Figure 2:**
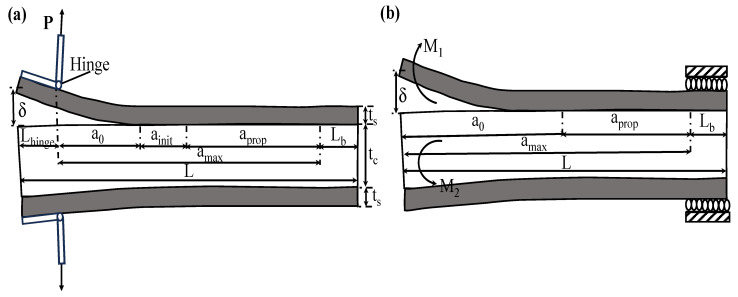
Schematics of different double cantilever beam (DCB) methods: (**a**) DCB and (**b**) DCB with uneven bending moment (specimen length (L), final debond (a_max_), hinge length (L_hinge_), initial debond length (a_o_), natural crack initiation (a_init_), crack propagation (a_prop_), intact length (L_b_), and bending moments (M_1_ and M_2_)).

**Figure 3:**
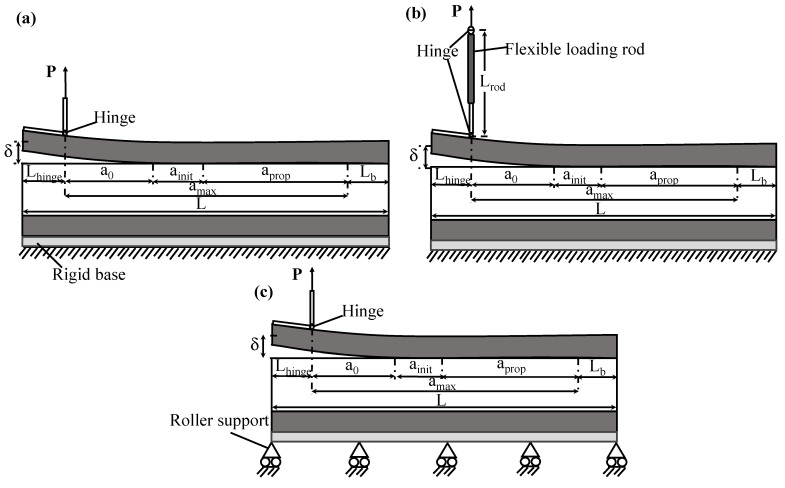
Prominent single cantilever beam test (SCB) methods: (**a**) single cantilever beam with rigid base, (**b**) SCB with long, flexible loading rod and (**c**) SCB with a rigid roller base (picture courtesy of [41]) (specimen length (L), final debond (a_max_), hinge length (L_hinge_), initial debond length (a_o_), natural crack initiation (a_init_), crack propagation (a_prop_), intact length (L_b_), and rod length (L_rod_)).

Without claiming completeness in obtaining dominant Mode I in sandwich panels, the most commonly utilised non-standardised and standardised test methods are classified into two: the cantilever beam methods (shown in Figure 2 and Figure 3), such as double cantilever beam (DCB) and single cantilever beam (SCB), and the peeling test, including the climbing drum peel test. A comparative summary of testing configurations, data reduction methods, and governing parameters is presented in Table 1. A more detailed overview of different methods for characterising Mode I interfacial fracture toughness, data reduction schemes and governing parameters is provided in the following subsections.

### 3.1. Double Cantilever Beam Methods

Mode I debonding is an interfacial failure mode in sandwich structures, in which the skin peels away from the core surface under tensile or opening-load conditions acting perpendicular to the skin/core interface. Under bending conditions, tensile normal stresses develop in the loaded skin and produce peel stresses at the skin/core interface, leading to local crack opening and progressive interfacial separation. However, the chances for attaining a dominant Mode I condition at the crack tips are challenging. Test methods designed to apply predominantly Mode I loading to the skin/core interface may still generate a secondary Mode II component when used for sandwich structures. According to Hutchinson and Suo [59,60,61], this behaviour is attributed to the elastic mismatch between the core and skins, as well as to asymmetries in specimen geometries and in the applied loading conditions. As a result, the interfacial crack experiences mixed-mode loading, which is commonly described in terms of a mode-mixity phase angle or a mixed-mode ratio. It provides a measure of the loading mode mixity and is defined based on the ratio of shear to opening energy release rates or stress intensity factors, depending on the formulation adopted.

The DCB method for sandwich panels is derived from the ASTM D 5528-21 standard [62] for monolithic polymer composite materials. The DCB configuration was modified for sandwich structures by Prasad and Carlsson [63] as shown in Figure 2a. Unlike the standard DCB specimen used for monolithic polymer composites, in sandwich structures, the pre-crack is not located at the mid-plane of the specimen thickness but is instead positioned along the interface between the core and the upper skin, as shown in Figure 2a,b. Although significant attempts have been made to modify the DCB method for application to sandwich structures [21,28,35,44,45,64,65], important difficulties persist. In particular, crack growth during the test causes a progressive rotation of the specimen around the bottom load application point [65,66], and bending moments are induced in the core due to the eccentric application of the load [23,43].

Hence, the DCB test method applied to sandwich structures involves a non-negligible Mode II component at the crack tip due to the asymmetric loading and material property mismatch [32,60,67]. It is widely accepted that a bi-material interface, such as the skin/core polymer interface in sandwich structures, results in coupling between normal and shear deformations [59,60,68,69]. Burlayenko et al. [28] examined the debonding behaviour of sandwich structures comprising glass fibre-reinforced epoxy skins and PVC foam cores using DCB and SCB configurations. Their experimental results, supported by 2D finite element analysis using plane strain elements, revealed significantly higher fracture toughness for the DCB method, which was attributed to pronounced Mode II fractions developed by the asymmetric loading. Moreover, they showed that shear stresses at the crack tip can induce crack kinking into the core. This result confirms earlier reports in which crack kinking in DCB specimens was associated with positive shear stresses (i.e., crack kinking into the core) at the crack tip. This arises from asymmetric loading conditions and elastic mismatch between the skin and core materials [32,63,70]. Based on these findings and their evaluation against the criteria for dominant Mode I sandwich testing procedures, the DCB configuration is deemed inappropriate for Mode I-dominated determination of the fracture toughness of the skin/core interface in polymer-based sandwich structures.

Numerical modelling has increasingly been used to support and validate experimental Mode I skin/core debonding characterisation in sandwich structures. Cohesive Zone Models (CZMs) and the Virtual Crack Closure Technique (VCCT) are among the most widely adopted approaches in the DCB configuration. This enables careful simulation of crack initiation and propagation while providing interface properties that are difficult to determine experimentally. Ramantani et al. [71] combined DCB testing with cohesive zone modelling to characterise skin/core debonding in carbon-fibre-reinforced polymer (CFRP)/PMI foam sandwich structures. Experimental fracture energies obtained using a compliance-based beam method (CBBM) were implemented in a finite element model employing interface cohesive elements. An inverse analysis based on matching numerical and experimental load–displacement responses was used to identify the interface cohesive strength. Furthermore, the VCCT was employed to verify the mode mixity at the crack tip, confirming that Mode I contributed approximately 97% of the total energy release rate. The excellent agreement between experimental and numerical R-curves demonstrated the capability of numerical methods to validate fracture characterisation procedures. Xue et al. [12] combined DCB testing with a nonlinear exponential cohesive zone model to investigate skin/core debonding in sandwich beams with CFRP hexagonal honeycomb cores. The numerical framework incorporated Timoshenko beam theory for the skins and Extended High-Order Sandwich Panel Theory (EHSAPT) for the honeycomb core. Comparison between theoretical predictions and experimental measurements demonstrated good agreement in fracture toughness evaluation. The identified fracture toughness values (0.22–0.38 kJ/m^2^) showed good agreement with experimentally measured values (0.21–0.59 kJ/m^2^). The study further revealed that although both normal and tangential tractions existed at the crack tip, the debonding process was primarily controlled by Mode I fracture. Similarly, Lee et al. [64] showed that pure Mode I fracture toughness of sandwich skin/core interfaces can be reliably measured using a modified double cantilever beam specimen with curvature-equalising metal plates to eliminate mode mixity. The interface exhibits a rising R-curve followed by a steady-state plateau; with the steady-state Mode I fracture toughness around 0.17–0.20 N/mm. The rising portion is attributed to complex fracture processes in the process zone involving interaction between the brittle skin and soft foam core. A trilinear cohesive zone model incorporating the R-curve successfully reproduces both load–displacement behaviour and crack growth, showing that a single constant toughness value is insufficient to capture the full delamination response.

Cantilever beam test methods, except the DCB with UBM, consist of applying a load *P* to debond a skin from the core of a sandwich specimen with a length (L), width (b), and total thickness (h). Before testing, a pre-crack of length a_0_ is introduced at the skin/core interface. The load (P) is applied through a piano hinge or connecting blocks, which may be adhesively or mechanically attached to the specimen. As the applied load and the load-point displacement (δ) increase, crack initiation occurs, followed by stable crack propagation along the interface. The fracture toughness, expressed in terms of the SERR, can be evaluated using several data reduction methods [71,72], including the modified beam theory (MBT) [8,50,62,73], the compliance calibration (CC) method [74,75], the compliance-based beam method (CBM) [71], and the elastic foundation analysis (EFA) method [35,76,77]. Kanninen [78] first developed the EFA approach for isotropic materials, and later extended it to orthotropic materials by Williams [79]. However, all these methods are valid only when the assumptions of linear elastic fracture mechanics (LEFM) are satisfied, particularly small deflections and linear elastic material behaviour [22,62].

Among these approaches, the MBT, originally proposed by Williams [79], is widely used due to its simplicity and reliability. For Mode I fracture of skin/core interfaces in DCB specimens, Shivakumar et al. [72] recommend MBT, which requires measurement of the specimen width *b*, applied load *P*, load point deflection *δ*, and crack length (a) during testing. The Mode I SERR is then evaluated using the MBT expression for $G_{I}$.

(1) $$G_{I}=\frac{3P\delta }{2b(a+\Delta )}\;J/m^{2}$$

where Δ is the effective crack length correction factor that accounts for root rotation and shear deformation at the crack tip. Here, Δ can be determined from the cube root of the compliance $C^{1/3}$, measured during testing, together with the corresponding crack length, a.

DCB with Uneven Bending Moments (DCB-UBM) is used for interfacial fracture testing with controlled mode mixity, primarily to measure Mode I dominant and mixed-mode (Mode I + Mode II) fracture toughness. The DCB-UBM specimen was first introduced by Sørensen et al. [36] for the determination of fracture toughness in monolithic composites. Later, Lundsgaard-Larsen et al. [39] extended it to sandwich structures to overcome the drawbacks of the existing DCB testing method for sandwich panels. The test configuration comprises the DCB specimen loaded with uneven bending moments. The fracture specimen principle of DCB-UBM in the sandwich panel is depicted in Figure 2b, in which the crack flanks or specimen edges are subjected to pure moments (*M*_1_ and *M*_2_). For instance, dominant Mode I loading is obtained by applying opening moments with $M_{1}>0$ and $M_{2}<0$. To ensure a constant mode mixity during fracture propagation, the two applied moments must be actively controlled such that the moment ratio (*MR*) remains constant throughout the test. When equal moments of opposite sign are applied (e.g., $M_{1}/M_{2}=-1$), the loading is dominated by crack opening in the normal direction, corresponding to Mode I fracture. Conversely, when moments of the same sign are applied, shear sliding along the crack interface prevails, leading to Mode II-dominated behaviour. Accordingly, the mode mixity can be prescribed by varying the ratio of the applied bending moments, $MR=M_{1}/M_{2}$. Since this ratio is kept constant throughout the test, the DCB-UBM configuration represents a steady-state fracture specimen. As a result, both fracture toughness and mode mixity, expressed as the phase angle ψ, remain constant during crack propagation, ensuring stable Mode I-dominated fracture conditions [46,80,81]. Hutchinson and Suo [59] defined the mode-mixity phase angle, ψ, as a parameter characterising the proportion of Mode II relative to Mode I loading at the crack tip. In this definition, ψ=0° corresponds to pure Mode I conditions, while ψ=90° denotes pure Mode II loading. Therefore, the phase angle serves as a practical parameter to describe deviation from ideal Mode I conditions in experimental configurations such as DCB and SCB tests.

A DCB-UBM test rig, capable of applying pure bending moments, is utilised to maintain a predominantly Mode I loading condition at the crack tip and to minimise Mode II contributions [46]. Several methods exist to maintain a constant moment ratio, *MR*, and each method is specific to the construction of the test rig. Hence, bending moments can be applied to the crack flanks through wire-and-roller systems [36], steel band-roller fixtures [82], rigid linkage mechanisms [83], or directly via independent torsional actuators [37,46], all of which represent different mechanical implementations of the same DCB-UBM loading principle. Berggreen et al. [37] developed a DCB-UBM test configuration, in which pure bending moments are applied directly to the crack flanks using independent torsional actuators. This setup enables controlled crack propagation along the glass-fibre skin/PVC H45 foam core interface. A constant mode-mixity was maintained throughout the experiments, enabling fracture toughness to be evaluated over a range of phase angles. In all cases, crack growth was confined to the skin/core interface. Under Mode I-dominated loading, the measured fracture toughness showed good agreement with values reported in previous studies employing alternative test configurations. A similar experimental configuration was utilised by Saseendran et al. [46] for fracture testing of aerospace-grade Nomex^®^- and Kevlar^®^-based honeycomb core of different grades of sandwich panels with carbon fibre-reinforced polymer skins. Mode I-dominated fracture toughness values measured along the longitudinal (L) and transverse (W) directions were compared across the different core densities (32, 64, and 96 kg/m^3^). The highest Mode I toughness (1511 J/m^2^) was obtained for the 96 kg/m^3^ core in the transverse direction, while the lowest value (872 J/m^2^) was recorded for the 32 kg/m^3^ core in the longitudinal direction.

The fracture characterisation is performed within the framework of Linear Elastic Fracture Mechanics (LEFM) [37,46]. When employing the DCB-UBM specimen for sandwich composites, it is critical to avoid competing failure mechanisms such as plastic yielding, damage dissipation, excessive deflections, and large rotations as these violate LEFM assumptions and compromise the validity of the measured fracture toughness. In particular, sandwich specimens with very thin skins are susceptible to large bending deformations with applied moments, which can invalidate the test results. To mitigate these effects, stiff reinforcement layers, typically high-strength steel, are bonded to the skins. These reinforcements reduce beam stresses and deflections while enabling reliable end-tab attachment using screws. The steel doublers are designed to remain fully elastic before crack propagation. The fracture toughness of a DCB-UBM specimen is evaluated using the J-integral, which under LEFM equals the energy release rate (*G*) [37,39]:

(2) G=J

The DCB-UBM specimen is modelled as three beams: (i) the debonded (upper) beam consisting of the top skin with or without a top doubler, contributing $J_{1}$; (ii) the substrate (lower) beam comprising the core, bottom skin, and optional bottom doubler, contributing $J_{2}$; and (iii) the intact base region ahead of the pre-crack, contributing $J_{3}$. The total J-integral is the sum of contributions from these three regions:

(3) $$J=J_{1}+J_{2}+J_{3}$$

Here, the index i= 1, 2, 3 corresponds to the debonded beam, substrate beam, and base beam, respectively. Each contribution $J_{i}$ is calculated from the applied bending moments $M_{i}$, the elastic modulus $E_{i}$, the beam stiffness terms $A_{i}$, $B_{i}$, $D_{i}$, and a geometric factor that depends on the layer thicknesses and their positions relative to the neutral axis ($y_{NA}$). The J-integral for each beam is given by

(4) $$J_{i}=\sum_{p=1}^{10}\frac{E_{i}M_{i}^{2}}{6\;(A_{i}D_{i}-B_{i}^{2}{)}^{2}}\left[A_{i}^{2}\left(y_{p-1}^{3}-y_{p}^{3}\right)-3A_{i}B_{i}\left(y_{p-1}^{2}-y_{p}^{2}\right)+3B_{i}^{2}\left(y_{p-1}-y_{p}\right)\right]$$

The extensional, coupling, and bending stiffnesses of each beam are determined from the material properties and geometry of each layer as $A_{i}=\sum_{k}E_{k}h_{k}$, $B_{i}=\sum_{k}E_{k}h_{k}\left(y_{k}-y_{NA}\right),\;and\;D_{i}=\sum_{k}\frac{E_{k}h_{k}^{3}}{12}+\sum_{k}E_{k}h_{k}(y_{k}-y_{NA}{)}^{2}$, where $E_{k}$ and $h_{k}$ are the elastic modulus and the thickness of the layer k, and $y_{NA}$ is the neutral axis of the corresponding beam.

Although the DCB with uneven bending moment (DCB-UBM) predominantly induces Mode I loading at the crack tip, the J-integral-based data reduction scheme, while providing a rigorous framework for evaluating fracture toughness with pure moments, relies on linear elastic beam theory. Consequently, it is highly sensitive to material properties, geometric parameters, and the determination of the neutral axis [37]. Moreover, the DCB-UBM fixtures require specialised moment application and have limitations in terms of achievable mode mixity and practical loading arrangements, indicating that the experimental setup is complex and sensitive to alignment and boundary conditions [37,80]. Owing to these criticalities, the applicability of the DCB-UBM configuration becomes restricted.

### 3.2. Single Cantilever Beam Methods

In search of a facile and dominant Mode I configuration, many researchers have developed and studied different variants of a single cantilever beam (SCB) [51], as shown in Figure 3. These setups offer simpler fixturing, improved stability during crack growth, and easier instrumentation compared with DCB-UBM.

Peel angles were varied by tilting the specimen at an angle, θ, and loading it with a vertical force, P, using the tilted sandwich debond (TSD) variant [84]. However, this configuration often introduces mixed-mode loading and instability in crack propagation, limiting its suitability for reliable Mode I characterisation. Based on extensive experimental and analytical studies by Adams [32] as well as Ratcliffe et al. [23], the SCB configurations have emerged as the most promising approach compared to the TSD and DCB methods for characterising Mode I fracture toughness. Burlayenko et al. [28] investigated the Mode I dominance of DCB and SCB (with rigid base) test methods for sandwich structures with varying material and geometrical parameters. Based on their findings, the SCB configuration proves to be significantly more effective in promoting Mode I-dominated fracture compared with the DCB test. Furthermore, in addition to the dominant Mode I characteristics, the SCB method shows only a limited propensity for crack kinking into the core, a behaviour also reported by several other authors [30,46,71]. The SERR for this configuration can be calculated using data reduction methods analogous to those employed in the DCB test [22,49].

The SCB test is a cantilever-type fracture configuration in which one unconstrained skin of a sandwich panel is debonded from the core while the opposite skin is constrained against a rigid base plate, as illustrated in Figure 3a. A load is applied to the free skin through a hinge or loading block, causing the crack at the skin/core interface to open primarily in Mode I. However, as the crack propagates during testing, the position of the load relative to the crack tip changes continuously. Consequently, the applied loading angle is not fixed but varies throughout the test. This variation generates shear stresses or horizontal load components within the core and gives rise to a Mode II contribution at the crack tip, which depends on the local crack-tip orientation [20,49,52,84]. Charalambides et al. [48] and Cantwell [49] reported that apart from the pure Mode I, a dominant Mode II contribution (37.6%) locally occurs in an SCB on a fixed base subjected to a bending moment. As a result, specific measures are implemented to maintain a fixed position of the crack tip relative to the load displacement axis of the testing machine throughout the test. Consequently, a modification of the conventional SCB configuration is necessary to attain a dominant Mode I condition at the skin/core interface. To attain this, two modified SCB approaches have been proposed in the literature: one utilises a movable base carrier (Figure 3b), while the other employs a long, flexible, and rotatable loading rod (Figure 3c).

In SCB with a movable base carrier setup, the sandwich specimen is attached to a rigid steel base plate mounted on a horizontally movable carriage, as shown in Figure 3b. The vertical load is applied at the free end of the cantilever through a hinge or loading block. Any horizontal components of the load cause movement of the sliding carriage, effectively eliminating shear forces at the crack tip. This ensures that the crack experiences primarily dominant Mode I opening, reduces the risk of crack kinking, and improves the accuracy and repeatability of fracture toughness measurements. This setup was developed by Cantwell and Davies [56] and employed by Glaessgen et al. [20]. In this approach, the horizontal movement of the base carrier ensures that the applied load acts purely in the vertical direction as any horizontal load components would result in displacement of the carrier. The same SCB testing configuration was also employed by Irven et al. [26,57] to investigate Mode I-dominated skin/core debonding in sandwich structures with carbon-fibre-reinforced epoxy (DGEBA) skins modified with CSR and silica nanoparticles and epoxy foam cores. While the sliding base carriage effectively minimises Mode II loading from the test setup, the results revealed that a negligible local Mode II component still arises, leading to crack propagation into the core. This behaviour was attributed to the mismatch in material modulus between the skin and the core, which promotes local shear stresses at the crack tip.

To reduce the complexity associated with the SCB configuration using a moving carriage, McGarva et al. [52,53] introduced an SCB setup with a long, flexible, and rotatable loading rod. In this arrangement, the long, flexible, and rotatable loading rod connects a hinge mounted on the SCB specimen skin to the testing machine, allowing the load to remain nearly vertical throughout testing, thereby minimising horizontal components (Mode II) and maintaining predominantly Mode I loading at the crack tip. A schematic representation of the SCB with a long, flexible load rod is displayed in Figure 3c. In this configuration, the SCB specimen is fixed to a rigid base plate, and the rotatable rod allows the vertical load to remain aligned with the crack opening throughout testing, accommodating deflections without introducing horizontal forces. Wilk [22] examined the applicability of an SCB with a long, flexible loading rod for the evaluation of Mode I-dominated debonding in carbon epoxy/aramid honeycomb sandwich panels under quasi-static and fatigue loading. Fracture toughness was evaluated using both MBT (1054 ± 50 J/m^2^) and the area method (1144 ± 47 J/m^2^), showing that the rotatable-rod SCB provides reliable Mode I characterisation with a simple fixture. In comparison, the area method is more robust for propagation due to difficulties in visualising crack lengths in honeycomb cores. The debond propagated predominantly by cohesive fracture within the core, occurring very close to the core/skin interface, just beneath the adhesive bond line. No evidence of crack kinking into the core was observed during propagation; however, the resulting fracture surface exhibited a rough and irregular morphology, indicative of non-uniform crack advance within the core material. A similar crack deviation into the core, attributed to the mismatch in skin and core moduli, was also reported by EASA (European Union Aviation Safety Agency) [51]. This deviation was observed even when a long, rotatable loading rod SCB configuration was employed, which is intended to maintain a nearly vertical load path throughout testing. This introduces a negligible Mode II component at the crack tip, promoting crack deflection into the core rather than maintaining a pure interfacial Mode I failure. However, by providing a considerably simpler test apparatus, the use of a long rotatable rod minimises Mode II loading and suppresses shear development in the core [23]. This advantage has motivated Ratcliffe et al. [23,43] and Adam [32] to adopt and further develop this setup for interfacial bonding characterisation and for the standardisation of Mode I interfacial fracture toughness testing of sandwich structures.

Although the challenges associated with the test setups are largely minimised by using an SCB configuration with either a moving carrier or a long, flexible loading rod, experimental results can still be significantly affected by specimen geometry, material properties, and local heterogeneities at the skin/core interface, such as variations in adhesive thickness, fibre distribution, or core cell structure. The influence of various geometric parameters, such as specimen geometry, skin thickness, core thickness, and core density, on the fracture behaviour of sandwich structures has been extensively studied by various researchers [70,85]. Maleki and Toygar [70] reported that core density significantly influences the measured SERR in GFRP sandwich structures with PVC foam cores tested using the SCB method. Their results showed that sandwiches with higher-density cores exhibit increased SERR values, indicating improved resistance to skin/core debonding. Moreover, Viana and Carlsson [85] provide further support for this observation as they also reported an increase in SERR with higher-density foam core sandwich structures, which inherently introduces a Mode II component. The increase in SERR with higher-density cores is attributed to the greater stiffness of the core, which reduces local deformation and ensures that more of the applied energy is directed towards opening the interface. Xue et al. [12] performed a parametric study to assess the influence of fracture toughness, characteristic length, skin thickness, and fracture-related parameters on the mechanical response of the sandwich structure. The study showed that higher fracture toughness increases fracture load, displacement, and interfacial strength, particularly at smaller characteristic lengths, while increasing the characteristic length reduces fracture load and interfacial stiffness, increases fracture displacement, and extends the damage zone. Thicker skins enhance bending and shear stiffness, resulting in higher interfacial strength and longer damage zones, whereas increasing core thickness slightly reduces interfacial strength with little effect on damage length. Similar findings of an increase in skin/core interfacial fracture toughness with the increase in skin thickness were reported by Alliyankal Vijayakumar et al. [41]. This trend was found to be similar across all the SCB configurations. It is found that the average fracture toughness for 5.4 mm skin specimens was 64.82% higher than that measured using 1.2 mm skin. Likely, Burlayenko et al. [28] found that specimens exhibiting negative phase angle values decrease with an increase in skin thickness. This trend is consistent across the full range of pre-crack lengths and material combinations. Therefore, thicker skins reduce the magnitude of the negative phase angle, helping maintain Mode I dominance across different crack lengths and material combinations. Adam [32], through both experimental and numerical studies, demonstrated that the use of a skin doubler to reduce skin deflection can alter the crack propagation path and affect the measured fracture toughness. Therefore, the use of skin doublers in SCB specimens with thin skins is not recommended. Denning et al. [86] demonstrated that failure mode strongly affects fracture toughness and resistance curves in sandwich structures. Core failure produces smooth, stable crack growth with low scatter, while pullout and adhesive failures show abrupt propagation and higher variability. Core type, cell size, and density influence the energy required for crack propagation, with higher-density cores requiring more energy. Also, changing the skin thickness can alter the failure mode of the material system, leading to significant differences in fracture toughness. Thicker skin specimens generally exhibit higher fracture toughness, as reflected in the resistance curves, although some systems show slightly rising or falling curves due to changes in failure mode and increased material nonlinearity. These findings emphasise the need to optimise core and skin properties in SCB specimens to achieve Mode I-dominated, reproducible measurements. Similar findings were also reported by several others [38,43,50,81]. Saseendran et al. [81] conducted a numerical parametric study using the Crack Surface Displacement Extrapolation (CSDE) method to investigate local mode mixity in sandwich structures, varying geometrical parameters (skin thickness and intact length) and material properties (skin-core modulus and Poisson’s ratio). They found that skin modulus, Poisson’s ratio, and thickness strongly influence the mixed-mode conditions, while the intact length significantly affects the phase angle (*ψ*), particularly for longer cracks in shorter specimens. Similarly, Burlayenko et al. [28] used finite element analysis to examine SCB and DCB polymer specimens and reported that both specimen types are highly sensitive to changes in material properties, geometrical parameters (skin thickness, initial debond and intact length), and boundary conditions, affecting both Mode I and Mode II fracture responses. Alliyankal Vijayakumar et al. [42] studied the influence of different intact (L_b_) and initial debond (a_0_) lengths on SCB Mode I fracture toughness. It was found that there exists a significant influence of the L_b_ and a_0_ on the Mode I fracture toughness of polypropylene (PP)-based thermoplastic sandwich panels. L_b_ and a_0_ dimensions lower than the proposed sizing constraints showed deviation from the dominant Mode I condition.

Apart from these geometrical influences, processing parameters and material (polymer)-dependent properties of the sandwich systems significantly affect the Mode I fracture toughness. Various polymer and process-dependent factors affecting Mode I characterisation in sandwich structures are tabulated in Table 2. Furthermore, average skin/core fracture toughness with different materials, manufacturing, geometrical, and testing methodologies are shown in Table 3. Processing temperature and pressure plays a critical role in determining the interfacial fracture behaviour of thermoplastic sandwich structures. As detailed in one-stage non-isothermal compression moulding process [41,42], an increase in press plate temperature from 30 °C to 50 °C was shown to enhance the measured Mode I fracture toughness by approximately 19% under dominant Mode I conditions in PP-based sandwich panels. This improvement is attributed to enhanced skin consolidation resulting from reduced cooling rates, which increases skin bending stiffness and modifies the global structural compliance in SCB configurations. Consequently, the energy release rate and the measured $G_{Icp}$ are directly affected. Also, a better interdiffusion of the molecular chain between the skin-core polymer resulted in cohesive fracture under Mode I loading. Ultrasonic welding parameters strongly influence interfacial fracture behaviour through their effect on polymer melting, flow, and interdiffusion at the interface of PP thermoplastic sandwich structures [55]. It was found that higher horn pressure (0.39 MPa) enhances interfacial contact and polymer chain mobility, resulting in cohesive failure within the core, indicating improved bonding. However, the lower 0.29 MPa resulted in a higher value for Mode I fracture than 0.39 MPa associated with crack propagation through the skin. Similarly, with respect to welding speed, a lower speed of 4 mm/s promotes cohesive failure in the core and therefore provides a more reliable measure of skin/core interfacial toughness. In contrast, at 5 mm/s, although higher fracture toughness values (~51%) are observed, failure occurs within the skin due to delamination. Thus, the measured values reflect the fracture resistance of the PP skin rather than the true skin/core interfacial bond strength. Furthermore, Trucillo et al. [54] observed that temperature variations across the steel mould during the manufacturing of PP sandwich panels through in situ physical foaming and skin/core bonding influenced the Mode I characterisation. Gas diffusion into PP molten polymer across the mould walls occurred at higher rate due to the increased temperature. This leads to variations in cell morphology alongside the mould walls of the polymeric foam core with low dense, larger cells, while the centre has a high dense, smaller cells. As a consequence of the Mode I test, the crack propagated through the weaker skin/core interface has a foam core with lower density and larger cells. This affected the Mode I fracture toughness of the in situ foamed and skin/core bonded sandwich panels. As reported by Viana et al. [85], resin or adhesive penetration into partially open core cells forms a dense interphase layer that resists crack propagation under Mode I loading, promoting failure within the core rather than along the interface. Additionally, the bimaterial interface drives crack kinking into the compliant core. Although not explicitly stated, this behaviour is governed by wettability and surface energy, which control resin infiltration and interfacial bonding quality. Similarly, brittle polymer foam structure and skin/core interface affecting the Mode I fracture behaviours were reported by [26,56,57]. These findings highlight that processing-induced variations in polymer properties can significantly influence fracture characterisation, demonstrating that measured toughness values reflect not only interfacial adhesion but also changes in structural stiffness and energy distribution. Therefore, the fracture toughness values alone cannot be relied upon to determine the performance of polymeric sandwich panels without considering mode of fracture.

The compiled results in Table 3 highlight that Mode I fracture toughness of sandwich structures exhibits substantial variability across material systems, manufacturing routes, and testing configurations. This variability is primarily attributed to differences in skin/core properties, interface quality, and process-induced defects, which strongly influence crack initiation and propagation behaviour. In general, well-consolidated and carefully processed sandwich systems tend to show comparatively lower scatter, whereas weakly consolidated and bonded architectures exhibit higher variability due to increased sensitivity to local defects and interfacial discontinuities. Among testing methodologies, SCB-based configurations often provide relatively consistent results. However, similar levels of scatter can also be observed in DCB-based configurations depending on crack path stability and interface quality, indicating that reproducibility is not governed by test geometry alone. Reproducibility remains strongly dependent on interface quality, specimen preparation, and load introduction method. Overall, the quantified scatter in the compiled dataset spans from very low values (~0.5–5% in highly controlled and well-consolidated systems) to moderate levels (~6–10%) and up to higher variability (>10–20%). This confirms that Mode I fracture toughness is not an intrinsic constant but a process- and configuration-dependent property. This reinforces the importance of reporting statistical parameters and experimental repeatability when comparing fracture toughness values across different studies.

Environmental conditions play a significant role in the Mode I fracture behaviour of sandwich composites. Factors such as moisture uptake, humidity exposure, thermal cycling, ultraviolet (UV) radiation, and long-term ageing can degrade the mechanical properties of skins, core materials, and skin/core interfaces [88,89]. Moisture absorption may cause plasticisation of polymer matrices and weakening of interfacial bonds [90]. Thermal cycling can induce residual stresses due to differences in the coefficients of thermal expansion between constituent materials. Furthermore, repeated temperature fluctuations may influence the degree of crystallinity through crystal reorganisation or secondary crystallisation, while prolonged thermal exposure can promote polymer chain relaxation, physical ageing, and, under severe conditions, chain scission. UV radiation may further induce photo-oxidative degradation, resulting in molecular chain scission, surface embrittlement, microcrack formation, and changes in crystallinity. These degradation mechanisms can reduce interfacial fracture toughness, alter crack propagation paths, and accelerate damage accumulation. Ding et al. [91] reported that combined solar radiation, moisture exposure, and hygrothermal cycling significantly degraded the mechanical performance of E-glass/PVC foam sandwich composites. This leads to approximately a 45% reduction in flatwise tensile strength and a transition in failure modes from core fracture to skin/core debonding, thus highlighting the strong influence of environmental ageing on interface durability. Despite their practical importance, environmental effects remain less extensively investigated than manufacturing and geometric parameters. This shows the need for further research aimed at understanding the long-term durability and fracture performance of sandwich structures under realistic service conditions.

In line with the dominance of opening-mode debonding in sandwich structures, Ratcliffe et al. [23,43] defined the requirements for a standardised test method to evaluate the reproducible and consistent Mode I-dominant skin/core fracture toughness in the SCB method. Building on these findings, specific constraints regarding specimen geometry and testing procedures were proposed to improve the repeatability and reliability of the SCB method. As a result of these collective efforts and the generally positive outcomes, a draft for an ASTM standard for the SCB method was developed and proposed to the ASTM D30 committee [32]. Subsequently, a large international round-robin testing programme was conducted by multiple research institutes and industrial laboratories worldwide [51]. These developments ultimately culminated in the publication of ASTM D8637/D8637M [34] in November 2025, a standard test method for Mode I dominant skin-to-core fracture toughness of sandwich constructions. This standard formalises the SCB configuration, specimen requirements, and data reduction procedures and defines Mode I dominance as having at least 90% of the total SERR in Mode I.

ASTM D8637/D8637M [34] proposes MBT (Equation (1)) as the data reduction method for determining crack initiation fracture toughness ($G_{Ici}$), and the area method (AM) for computing crack propagation fracture toughness. The propagation fracture toughness ($G_{Icp}$) measured utilising the AM is given as

(5) $$G_{Icp}=\frac{dU}{dA}$$

where dA = incremental crack area (mm^2^), b = specimen width (mm), $a_{1}$ = initial crack length (mm), and $a_{2}$ = final crack length after increment (mm).

(6) $$dA=b\;(a_{2}-a_{1})$$

Several authors consider the average propagation fracture toughness ($G_{Icp}$) as the value for fracture toughness in sandwich structures [23,41,42,55]. Wilk [22] conducted experiments that yielded similar results for the propagation toughness (G*_Icp_*) in SCB specimens, as evaluated using the two proposed data reduction methods (MBT and AM), shown in Table 3. The coefficient of variation of the measured Mode I fracture toughness was 4.1% for the AM and 4.7% for the MBT approach, indicating excellent repeatability and low experimental scatter. However, accurate measurement of debond length during propagation remains challenging, particularly due to the honeycomb core structure, which affects visual tracking. Consequently, the AM is preferred as it relies only on initial and final crack length measurements. In contrast, the MBT approach is more sensitive to crack length measurement and may be influenced by run-arrest propagation behaviour, often yielding more conservative results. Drake et al. [92,93] investigated SCB testing for sandwich structures and compared different data reduction methods for opening-mode fracture toughness. The study showed that the AM is commonly used but requires periodic unloading, making it impractical for instantaneous evaluation. In contrast, the MBT showed deviations at large displacements due to nonlinear compliance effects and empirical crack-root rotation corrections. A numerical virtual crack-closure technique (VCCT) demonstrated good agreement with experimental results and provided an alternative J-integral-based approach for more accurate and instantaneous fracture toughness evaluation. Moreover, a similar propagation fracture toughness was reported with both MBT and AM.

**Figure 4:**
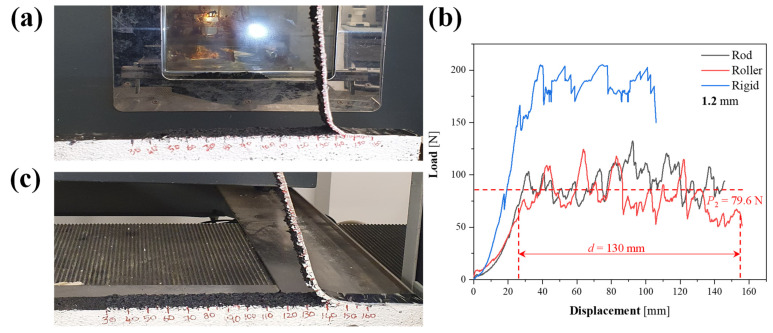
(**a**) Skin peeling under SCB conditions with the roller base (90°), (**b**) load–displacement curve with compliant skin specimens (1.2 mm skin thickness) for different SCB setups and (**c**) SCB skin peeling with the long, rotatable loading rod (<90°) (picture courtesy of [41]).

The two data reduction methods reported in the standard (Equations (1) and (5)) calculate the dominant Mode I fracture toughness in a sandwich structure utilising the linear elastic fracture mechanics. Therefore, their applicability is limited to specimens that demonstrate linear fracture responses, and no established methodology currently exists for specimens with compliant or highly deformable skins that exhibit non-linear loading behaviour. A comparative analysis among various SCB setups for determining the dominant Mode I in sandwich panels was performed by Alliyankal Vijayakumar et al. [41]. Both the SCB with the roller base and the long, rotatable loading rod configurations provide a dominant Mode I loading condition at the crack tips for stiffer and compliant skin specimens. Consequently, both configurations yield a similar average fracture toughness for stiffer skin specimens (4910 ± 416 J/m^2^ with long-flexible rod and 5216 ± 454 J/m^2^ with a roller base). The SCB with the rigid base setup showed a mixed-mode behaviour, exhibiting an average fracture toughness of 4087 ± 324 J/m^2^. Moreover, a near 90° peeling effect was observed for the SCB configuration with a roller base when testing compliant skin specimens exhibiting non-linear load–displacement behaviour, as shown in Figure 4a. As a result, a sawtooth load–displacement curve was observed as illustrated in Figure 4b. In contrast, the long, rotatable loading rod configuration induced a peeling angle lower than 90°, promoting crack kinking into the core rather than propagation along the skin/core interface, as depicted in Figure 4c. This led to a lower value for fracture toughness (1727 ±168 J/m^2^) compared to the roller-base configuration (2552 ±230 J/m^2^). These values were measured using MBT, assuming a linear elastic loading behaviour. With the roller base configuration, crack propagation is confined to the skin/core interface, thereby ensuring predominantly Mode I conditions at the crack tip. This observation motivated the modification of the climbing drum peel (CDP) equation for a compliance-independent Mode I fracture toughness expression. The developed propagation Mode I fracture toughness equation (G*_Icp_*) for SCB specimens exhibiting non-linear behaviour in conjunction with the roller base setup is given as

(7) $$G_{\mathrm{Icp}}=\frac{{(P}_{2})\;d}{b\;D}\;J/m^{2}$$

**Figure 5:**
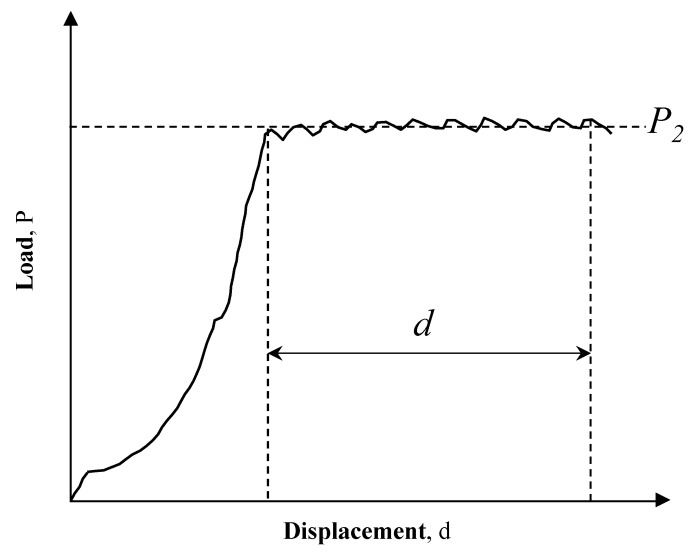
The schematics of the load–displacement curve for compliant skin specimens.

*P*_2_ is accounted for by the average force required to completely peel off the skin from the core surface during crack propagation. *d* refers to the displacement of the load frame, *b* is the width of the specimen, and *D* signifies the total crack length or the length of the peeled skin. The parametric calculations for measuring the G*_Icp_* were schematised in the load–displacement curve indicated in Figure 5. This compliance-independent data reduction method offers more reliable results for compliant skin specimens than those obtained using the MBT. In this approach, micro-cracks in the flexible skin material can affect test results. This method measured a fracture toughness 43% higher than the MBT method.

Likewise, Alliyankal Vijayakumar et al. [54,55] utilised two data reduction schemes to compute the Mode I fracture toughness of SCB specimens exhibiting non-linear fracture conditions. These investigations were performed on a full thermoplastic PP-based sandwich system. This was based on one assuming a linear elastic fracture mechanics (LEFM) using MBT (using Equation (1)) and another employing the climbing drum peel (CDP) expression (using Equation (7)). The data reduced using the compliant-independent CDP expression exhibit higher fracture toughness than those reduced using the compliant-dependent MBT. The average fracture toughness reduced by 44–47% with MBT when compared to the CDP method. In the MBT approach, the specimen compliance (*C*), which is directly related to structural stiffness, significantly influences the calculated Mode I fracture toughness. In particular, MBT tends to underestimate the skin/core debonding toughness for specimens with lower stiffness or non-linear loading, especially when a ductile interface is present. These approaches are effective for assessing Mode I fracture toughness, for comparing processing parameters and for optimisation purposes.

SCB dimensional constraints are proposed in ASTM D8637/D8637M [34] for the initial debond and intact lengths (shown in Equation (11)), aimed at ensuring dominant Mode I bending conditions. The initial debond (a_0_) and intact specimen lengths (L_b_), as denoted in Figure 2, proposed by the standard to reduce the shear deformation are as follows:

a_0_ ≥ a_0min_ = 2.7/λ (8)

L_b_ ≥ L_bmin_ = 2.7/λ (9)

*λ* is given as the ratio of the stiffness of the elastic foundation to the bending stiffness of the top skin and is given as

(10) $$\lambda ={\left[\frac{3\;k}{{ts}^{3}\;E_{fs}b}\right]}^{\frac{1}{4}}$$

Here, *b* represents the width of the specimen, while t_s_ and E_fs_ denote the thickness and flexural modulus of the skin, respectively. *k* is the elastic foundation modulus and is related to the z-direction modulus of the core material. The optimal value for *k* is given as E_cz_b/t_c_ by Li et al. [76] to minimise the shear and provide in the expressions proposed by Ratcliffe et al. [23]. E_cz_ is Young’s modulus of the core along the thickness direction, and t_c_ is the core thickness. Then, the final expression for computing the values for a_0_ and L_b_ is derived by substituting Equation (10) with the above expression for *k* into Equations (8) and (9), resulting in the following:

(11) $$a_{0}\;\geq \;a_{0\min}\;=\;L_{b}\;\geq \;L_{\mathrm{bmin}}\;=\;2.7\;{\left[\frac{tc\;{ts}^{3}\;E_{fs}}{3\;E_{cz}}\right]}^{\frac{1}{4}}$$

The a_0_ and L_b_ are kept above or equal to the minimum value according to Equation (11) to reduce the shear stress at the crack tips. However, the application of Equation (11) results in a significantly larger specimen dimensions for SCB configurations with higher skin stiffness or thickness. This requirement restricts the practical applicability of the standard, particularly for stiff sandwich systems due to increased material and experimental limitations.

To solve this, Alliyankal Vijayakumar et al. [42] proposed two equations for a_0_ and L_b_, where *k* is expressed in terms of 2E_cz_b/t_c_ and 2E_cz_b/t_s_, as suggested by Aviles et al. [35] and Quispitupa et al. [94], respectively. Substituting these values for *k* in Equation (10), and subsequently into Equations (8) and (9), gives the expressions for a_0_ and L_b_ as

(12) $$a_{0}\;\geq \;a_{0\min}=L_{b}\;\geq \;L_{\mathrm{bmin}}=2.7\;{\left[\frac{tc\;{ts}^{3}\;E_{fs}}{6\;E_{cz}}\right]}^{\frac{1}{4}}$$

(13) $$a_{0}\;\geq \;a_{0\min}=L_{b}\;\geq \;L_{\mathrm{bmin}}=2.7\;{\left[\frac{{ts}^{4}\;E_{fs}}{6\;E_{cz}}\right]}^{\frac{1}{4}}$$

It was observed that the SCB sizing dimensions obtained by Equations (12) and (13), with lower dimensions, yielded average Mode I fracture toughness values comparable to those obtained using the dimensions recommended by ASTM D8637/D8637M [34] (Equation (11)). Nonetheless, more stable crack propagation into the core and parallel to the skin/core interface was observed for specimens designed according to Equation (11). Comparatively, the SCB based on Equations (12) and (13) showed deeper crack propagation into the core. Therefore, these Equations (12) and (13) can be considered for measuring a_0_ and L_b_ in certain situations where the testing dimensions are limited. Otherwise, Equation (11) is considered the optimum sizing criterion for a_0_ and L_b_ to maintain a dominant Mode I condition at the crack tip. However, it was also found that deviations from the dimensioning criteria specified in Equations (11)–(13) leads to a variation from dominant Mode I behaviour. Table 4 tabulates the summary of equations used for the recommended SCB test configurations for characterising Mode I fracture toughness in sandwich structures.

### 3.3. Climbing Drum Peel Test

When the skin is compliant or flexible and shows non-linear load–displacement behaviour, conventional SCB or DCB-based fracture toughness methods become unreliable. This is because these methods rely on LEFM assumptions, which are violated when the polymer-skin undergoes large deformations, plasticity, or significant bending non-linearity. In such cases, the energy release rate (SERR, $G_{I}$) cannot be accurately determined using standard beam theory or compliance-based approaches.

To overcome this limitation, a peeling test can be used as an alternative method for quantifying skin/core fracture toughness. Peeling tests for polymer-based sandwich structures were primarily derived from the determination of the peel strength of metal–metal bonds [95,96]. In addition to metal–metal bonds, many other material combinations require interfacial bonding evaluation. These include rigid-rigid systems such as welded thermoplastic composites, hybrid metal–composite structures, and adhesively bonded metals [97,98,99,100,101]; flexible-rigid systems such as coatings, thin polymer films, and printed circuit boards on rigid substrates [102,103,104]; and flexible systems such as multilayer foils and packaging materials [105,106,107].

In a peeling test, the skin or the top adherent is peeled away from the core or substrate at a controlled angle (typically 90° or 180°) while measuring the peeling force. Unlike the SCB approach, this method does not require linear elastic behaviour of the skin, making it suitable for flexible, ductile, or polymeric skins. The fracture toughness is then determined from the measured peel force using an energy balance approach rather than beam bending theory [108]. The work done by the peeling force is related directly to the interfacial fracture energy, accounting for both fracture and any plastic deformation in the skin [109]. This makes the peeling test more representative of real debonding mechanisms in compliant sandwich structures.

**Figure 6:**
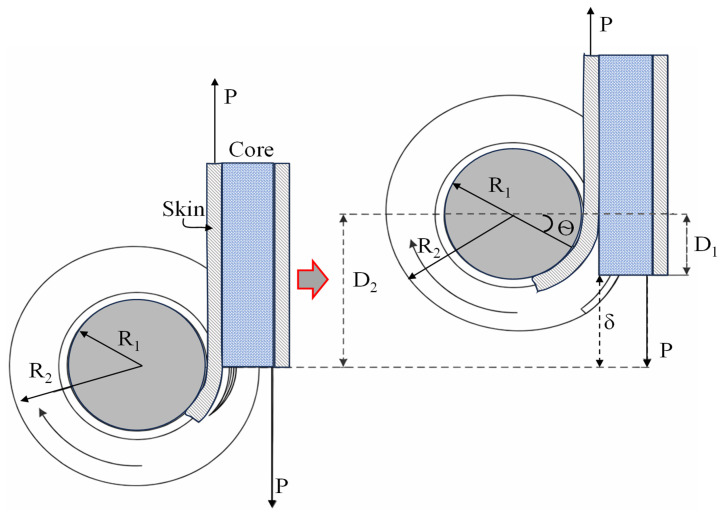
Schematic illustration of the climbing drum peel (CDP) test, showing the initial configuration (**left**) and the configuration after peeling a skin length D_1_ (**right**) (adapted from [8,43]).

In the context of sandwich structures, the available literature on peel-based interfacial fracture testing is extremely limited. To date, the climbing drum peel test appears to be the only peel configuration specifically applied to sandwich composites. Consequently, in this review, the climbing drum peel test is considered the sole relevant peel-based method for evaluating skin/core interfacial fracture toughness in sandwich structures. The climbing drum peel (CDP) test was originally standardised in the 1960s as ASTM D1781 [58]. It has since been widely used to characterise delamination resistance and skin/core interfacial bonding in sandwich structures, including thermoplastic sandwich systems. In this method, the upper skin of the sandwich panel is bonded to a rotating drum comprising two concentric sections with different radii, as shown in Figure 6. The skin is secured to the inner drum section of radius R_1_, while tension straps are attached to the outer section of larger radius R_2_ (R_2_ > R_1_). Both the straps and the sandwich specimen are clamped on opposite sides of a uniaxial testing machine. As the machine applies tensile load to the straps, a torque is transmitted to the drum, causing it to rotate along the surface of the sandwich specimen. This controlled rotation progressively peels the skin away from the core, enabling stable crack propagation under approximately constant load conditions. In its standard form, the CDP test provides a measure of peel strength, expressed as force per unit width, and allows quantitative comparison of results only when the specimen and drum geometry remain unchanged. However, Nettles et al. [29,110] proposed a method to determine the Mode I fracture toughness from CDP tests, building on the work of Okada and Kortschot [111].

In the CDP setup, the kinematic constraints cause the instantaneous crack length $D_{1}$ to depend directly on the crosshead displacement δ and the drum radii $R_{1}$ and $R_{2}$, as expressed by

(14) $$D_{1}=\frac{R_{1}\;\delta }{R_{2}-R_{1}}$$

**Figure 7:**
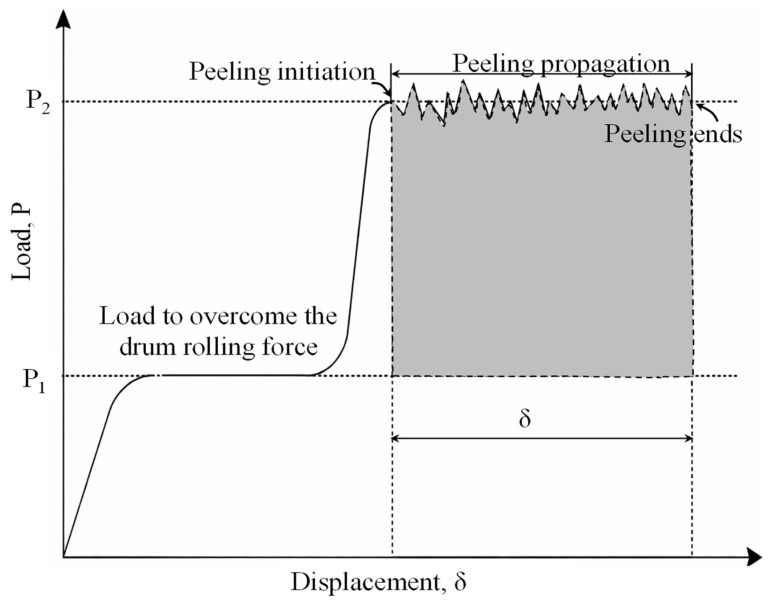
Sketch of the experimental load–displacement of the climbing drum peel test (adapted from [8]).

During the peeling process, two distinct load stages are observed as indicated in Figure 7. Initially, the skin is wrapped around the inner drum of the radius $R_{1}$ using the pre-crack region, without any skin/core debonding, generating a load $P_{1}$. This is followed by the initiation and stable propagation of the skin/core debond under a nearly constant load $P_{2}$. The work done during the peeling process is indicated as a grey area in Figure 7, can be translated into the fracture toughness by relating the energy to the peeled surface area, the specimen width b, and the crack length $D_{1}$. The SERR (*G_I_*) in the CDP test can be calculated as

(15) $$G_{I}=\frac{(P_{2}-P_{1})\;\delta }{b\;D_{1}}$$

Using this approach, the debonding of carbon fibre-reinforced epoxy skins from glass/phenolic honeycomb cores was experimentally evaluated. Comparisons with conventional DCB tests showed that both methods yielded similar $G_{I}$ values. However, the CDP test offers a clear advantage in terms of ease of execution and practical handling. Moreover, Adams et al. [32] also performed a comparative analysis between the CDP test and the SCB with a rigid base method for honeycomb-based sandwich panels. They carried out Mode I fracture toughness measurements using different drum peel radii, 2 in. (as specified in ASTM D1781 [58]), 4 in., and 6 in., along with various skin thicknesses (3, 6, and 9 plies). Their findings showed that the SERR derived from the SCB test is sensitive to changes in skin thickness, whereas the CDP test generally produces SERR values that are less dependent on thickness. However, they also observed that modifying the roller diameter in the CDP test resulted in varying SERR values. This variation was attributed to potential conformity issues in roller peel tests, particularly because there is no predefined alignment between the bending arm and the roller. Furthermore, the CDP method assumes perfect contact between the bending arm and the drum during SERR calculation, a condition that may not always be achieved experimentally.

Restrictions on utilising the CDP test are primarily associated with the mechanical behaviour of the skin and the resulting failure mode of the sandwich structure. The skin cannot be excessively thick or stiff as it must be able to conform to the curvature of the drum to ensure meaningful loading conditions. In addition, the test is most appropriate when interfacial debonding between the skin and the core is the dominant failure mechanism; if fracture instead occurs in the core or within the skin, the measured SERR no longer represents a true characterisation of the bondline fracture toughness. Finally, the skin must have adequate tensile strength to avoid premature tensile rupture during loading, which would invalidate the test outcome.

## 4. Conclusions

The present review critically examined a range of experimental methods for the accurate characterisation of dominant Mode I fracture behaviour in polymer-based sandwich structures, with particular emphasis on the applicability and limitations of ASTM D8637/D8637M. A comparative assessment of DCB, SCB, and CDP configurations highlighted their ability to achieve dominant Mode I conditions, as well as their experimental complexity and practical limitations. Furthermore, the influence of polymer-dependent response, processing effects, and fracture behaviour on the Mode I characteristic was detailed.

The analysis shows that conventional DCB configurations are inherently affected by mixed-mode conditions, making them unsuitable for accurate Mode I characterisation. Although modified DCB-UBM configurations can effectively reduce these effects, their experimental and analytical complexity limits their broader applicability. In contrast, SCB configurations provide a more practical and adaptable approach. However, maintaining dominant Mode I conditions strongly depends on boundary conditions, with a rigid base inevitably introducing mixed-mode effects, proving ineligible to maintain Mode I conditions. On the other hand, the SCB configuration with roller-base and long, flexible, and rotatable loading systems proves more effective in maintaining dominant Mode I conditions. Considering design simplicity, the approach with the long, flexible, and rotatable loading rod with sizing requirements was adopted in ASTM D8637/D8637M for measuring dominant Mode I fracture toughness. The CDP test is suitable for sandwich structures with thin, compliant skins exhibiting non-linear behaviour. However, its accuracy is affected by drum diameter and contact uncertainties. Thus, while it is useful for parametric and comparative studies, further refinement is needed for reliable evaluation.

A key limitation of ASTM D8637/D8637M is its restriction to linear elastic fracture behaviour, making it unsuitable for sandwich structures with non-linear responses. In addition, the standard imposes stringent specimen sizing requirements, particularly for intact and initial debond lengths (Equation (11)), which often result in impractically large specimens and increased material and experimental constraints. Furthermore, the prescribed SCB configuration does not fully account for variations in boundary conditions that can significantly influence crack tip loading.

The study also highlights the critical influence of geometrical and material parameters on fracture behaviour. It is demonstrated that alternative reduced-dimension specimen sizing (Equations (12) and (13)) can yield fracture toughness values comparable to those prescribed in the standard, offering greater flexibility in specimen design while reducing experimental constraints. The SCB geometries not complying with these sizing constraints show shear deformations at the crack tip, thereby overestimating fracture toughness values. The increasing skin thickness enhanced bending stiffness, promoted more linear elastic behaviour, and increased measured fracture toughness. In terms of data reduction, although the standard prescribes MBT for initiation and AM for propagation fracture toughness, both methods were found to yield comparable propagation toughness under linear-elastic conditions, indicating flexibility in their application. For the SCB exhibiting non-linear behaviour, the modified CDP concept (Equation (7)) with the roller-base supports provides a more suitable framework. However, future investigations are essential in analysing limitations of the compliance-independent data reduction method adopted in the modified CDP concept. Additionally, the Mode I interfacial fracture behaviour in polymer-based sandwich structures is strongly governed by the interplay between processing conditions, polymer properties, environmental exposure and interface quality.

Overall, this review identifies key limitations in the existing standard and provides new insights into boundary-condition design, specimen-sizing flexibility, and data reduction strategies. Future work should focus on developing unified testing methodologies capable of accurately capturing non-linear fracture behaviour, adapting existing test methods, integrating experimental and numerical approaches, and refining standardised procedures to accommodate a wider range of material systems and realistic service conditions.

## Figures and Tables

**Table 1:** Summary of experimental methods and parameters for Mode I fracture characterisation in polymer-based sandwich structures (fracture behaviours are tabulated based on the literature studies conducted on thermoplastic sandwich specimens exhibiting excellent skin/core interfacial adhesion).

Category	Method/Parameter	Key Features	Advantages	Fracture Behaviour	Impact on Characterisation	Applicability
Testing Configuration	DCB [8,21,35,44,45]	Symmetric beam loading	Simple concept, widely used	Crack kinks deeper into the core (Mode II)	Mixed-mode effects due to rotation and eccentric loading	Not suitable for pure Mode I in sandwich
	DCB-UBM [36,37,38,39,46]	Uneven bending moments	Reduces Mode II contribution	Crack propagates closer to the interface (dominant Mode I)	Complex setup and data reduction	Research-level applications
	SCB (rigid base) [8,41,47,48,49]	Single arm loading with fixed base	Simple setup, asymmetric loading lower vs. DCB	Excessive Mode II propagates the crack along the core and skin/core interface	Load angle variation → mixed-mode	Limited Mode I dominance
	SCB (long, flexible, rotatable rod) [8,22,23,41,43,50,51,52,53]	Controlled boundary conditions	Achieves dominant Mode I	Cracks propagate closer and parallel to the interface (dominant Mode I)	Requires careful setup design and sensitivity to specimen geometry	ASTM D8637 [34]
	SCB (roller base) [8,26,41,42,49,54,55,56,57]	Single arm loading with movable base	Achieves dominant Mode I	Propagates cracks closer and parallel to the interface (dominant Mode I)	Requires careful setup design and sensitivity to specimen geometry	Suitable for linear/non-linear loading, Basics of ASTM D8637 [34]
	CDP [8,29]	Peel-based loading	Suitable for thin/compliant skins	Propagates cracks closer or along the interface (dominant Mode I)	G_Icp_ changes with drum diameters	For parametric optimisation purposes [41,54,55], ASTM D1781 [29,58]
Data Reduction Methods	MBT	Compliance-based (LEFM)	Suitable for initiation and propagation of toughness	-	Sensitive to crack length, conservative	Linear elastic systems
	AM	Energy-based (dU/dA)	Simple, robust for propagation	-	Requires unloading, not instantaneous	Linear elastic systems
	J-integral/VCCT	Numerical methods	Handles non-linear behaviour	-	Requires modelling	Advanced analysis
	CDP/modified CDP expression	Peel mechanics	Suitable for non-linear behaviour	-	Less standardised and skin micro-cracks during the loading are neglected	Compliant skins, use with SCB–roller base and CDP setups
Geometrical/Material Parameters	Skin thickness/modulus	Controls bending stiffness	Thickness increase promotes higher stiffness	-	Low values affect fracture toughness	Critical design parameter
	Core density/modulus	Affects stiffness mismatch	Influences crack propagation	-	Affects toughness values	Critical design parameter
	Initial debond length	Crack starter	Controls energy release	-	Large sizes required in ASTM	Design constraint
	Intact length	Load transfer region	Ensures Mode I dominance	-	Leads to large specimens	Design limitation

**Table 2:** Polymer-dependent factors influencing Mode I fracture toughness characterisation in sandwich structures.

Category	Method/Parameter	Influence on Fracture Behaviour	Impact on Characterisation (Mode I Tests)	Typical Effect on Measured Toughness
Polymer properties	Crystallinity (thermoplastics)	Influences stiffness and crack resistance	Alters crack path and energy absorption	Higher crystallinity → higher toughness
	Intermolecular diffusion (fusion bonding)	Controls interfacial strength in thermoplastics	Dependent on temperature and pressure	Improved diffusion → higher toughness
	Surface energy/wettability	Affects bonding quality during processing	Influences repeatability of results	Poor wetting → lower toughness
	Cell structure (foam/honeycomb morphology)	Influences crack deviation into core	Affects failure mode interpretation	Irregular cells → scatter in results
Processing parameters	Skin/core consolidation pressure	Controls interfacial adhesion, diffusion/penetration of polymer chains and void content	Influences bonding quality	Requires careful optimisation for improved toughness
	Processing temperature	Governs melting, curing, and diffusion	Affects interfacial integrity	Optimal temperature → maximum toughness
	Cooling rate	Influences residual stresses, void formation and crystallinity	Affects crack propagation behaviour	Fast cooling → residual stress → affects measurements

**Table 3:** Mode I skin/core debonding toughness for various material, skin thickness, manufacturing and testing methodologies (Polypropylene: PP, Diglycidyl Ether of Bisphenol A: DEBA, Polyamide: PA, Polymethacrylimide: PMI, Phenylethynyl-Terminated Imide 5: PETI-5, Polyethylene Terephthalate: PET, Polyvinyl Chloride: PVC).

Polymer System	Skin Thickness	Manufacturing Method	Mode I Testing Set-Up	Data Reduction Scheme	Average Fracture Toughness (J/m^2^)
PP e-glass skin and PP foam core	5.4, 3 and 1.2 mm	Compression moulding (CM)	SCB with roller	MBT	5216 ± 454 (5.4 mm), 3678 ± 231 (3 mm) and 2552 ± 230 (1.2 mm) [41,42]
PP e-glass skin and PP foam core	5.4, 3 and 1.2 mm	CM	SCB with long flexible rod	MBT	4910 ± 461 (5.4 mm), 3624 ± 300 (3 mm), and 1727 ± 168 (1.2 mm) [41]
PP e-glass skin and PP foam core	5.4, 3 and 1.2 mm	CM	SCB with rigid base	MBT	4087 ± 324 (5.4 mm), 2371 ± 214 (3 mm) and 1080 ± 80 (1.2 mm) [41]
PP e-glass skin and PP foam core	1.2 mm	CM, continuous ultrasonic welding (USW) and in situ foaming (IF)	SCB with roller	Modified CDP	3645 ± 417 (CM), 4641–6940 (USW) and 3205 ± 626 (IF) [41,54,55]
Cf/DGEBA skin and epoxy foam core	3.2 mm	Resin infusion	SCB with roller	MBT	151–190 [26,57]
Glass epoxy skin and Al honeycomb and Al foam core	1.5 mm (10 ply) + 6 mm (40 ply)	Autoclave curing + secondary bonding	SCB with rigid	MBT	166–1608 [87]
Cf/epoxy skin and aramid honeycomb core	-	Autoclave	SCB with long, flexible rod	AM and MBT	1144 ± 47 (AM) and 1054 ± 50 (MBT) [22]
Glass fibre/PA12 skins + PMI (Rohacell 110 IG) foam core	0.8 mm	Compression moulding	SCB with long, flexible rod	MBT	610 ± 3 [52]
PETI-5 Carbon fibre (cf) skin and PETI-5 cf honeycomb core	3.3 mm	Adhesive bonding using PETI-5 film	DCB	MBT	1820 ± 340 [65]
Glass fibre/PA6 skin and PET foam core	1 mm	Adhesive bonding using polyolefin film	DCB	MBT	184.4 ± 12.4 [64]
Cf/epoxy skin and CFRP Honeycomb Core	1 mm	Adhesive bonding	DCB	MBT	100–2190 [44]
E-glass vinylester skin and PVC foam core	1.7 mm	Vacuum-assisted resin transfer moulding	DCB	MBT	693–1008 [66]
Carbon/epoxy skin and glass/phenolic honeycomb	3 ply	Adhesive bonding	DCB and CDP	MBT and CDP	1201–1384 (DCB) and 1208–1383 (CDP) [29]
CFRP/epoxy skins and Nomex or Kevlar honeycomb core	0.35 and 1.4 mm	Autoclave co-curing	DCB-UBM	J-integral based LEFM	1250–1550 (0.35 mm) and 900–1150 (1.4 mm) [46]

**Table 4:** Summary of equations of the recommended SCB test configurations for measuring specimen design criteria and fracture toughness in Mode I skin/core interfacial fracture characterisation.

Testing Configurations and Loading Conditions	Parameters	Equation	Data Reduction Methods/Specification
SCB with roller base or long, flexible loading rod with linear elastic loading condition	Fracture toughness initiation	$G_{Iini}=\frac{3P\delta }{2b(a+\Delta )}$	MBT
	Fracture toughness propagation	$G_{Icp}=\frac{dU}{b\;(a_{2}-a_{1})}$	Area
	Initial debond (a_0_) and intact (L_b_) specimen length	a_0_ ≥ a_0min_ = L_b_ ≥ L_bmin_ = 2.7 ${\left[\frac{tc\;{ts}^{3}\;E_{fs}}{3\;E_{cz}}\right]}^{\frac{1}{4}}$	Higher sizing dimensions
		a_0_ ≥ a_0min_ = L_b_ ≥ L_bmin_ = 2.7 ${\left[\frac{tc\;{ts}^{3}\;E_{fs}}{6\;E_{cz}}\right]}^{\frac{1}{4}}$	Intermediate sizing dimensions
		a_0_ ≥ a_0min_ = L_b_ ≥ L_bmin_ = 2.7 ${\left[\frac{{ts}^{4}\;E_{fs}}{6\;E_{cz}}\right]}^{\frac{1}{4}}$	Smaller sizing dimensions
	Rod length	L_rodmin_ = 2L, L is the specimen length	-
SCB with roller base for non-linear loading behaviour	Fracture toughness propagation	*G_Icp_* = $\frac{{(P}_{2})d}{b\;D}$	Modified CDP

## Data Availability

The original contributions presented in the study are included in the article; further inquiries can be directed to the corresponding authors.

## Abbreviations

The following abbreviations are used in this manuscript

SCB	Single Cantilever Beam
DCB-UBM	Double Cantilever Beam with Uneven Bending Moment
DCB	Double Cantilever Beam
CDP	Climbing Drum Peel
SERR	Strain Energy Release Rate
TSD	Tilted Sandwich Debond
LEFM	Linear Elastic Fracture Mechanics
MBT	Modified Beam Theory
AM	Area Method
G_Icp_	Mode I Fracture Toughness Propagation
